# Ten simple rules to run a successful BioHackathon

**DOI:** 10.1371/journal.pcbi.1007808

**Published:** 2020-05-07

**Authors:** Leyla Garcia, Erick Antezana, Alexander Garcia, Evan Bolton, Rafael Jimenez, Pjotr Prins, Juan M. Banda, Toshiaki Katayama

**Affiliations:** 1 ZB MED Information Centre for Life Sciences, Cologne, Germany; 2 Department of Biology, Norwegian University of Science and Technology, Trondheim, Norway; 3 Bayer CropScience SA-NV, Diegem, Belgium; 4 BASF, Ludwigshafen am Rhein, Germany; 5 National Center for Biotechnology Information, National Library of Medicine, National Institutes of Health, Bethesda, Maryland, United States of America; 6 Alzheimer's Research UK, Cambridge, United Kingdom; 7 University of Tennessee Health Science Center, Memphis, Tennessee, United States of America; 8 Georgia State University, Atlanta, Georgia, United States of America; 9 Database Center for Life Science, Chiba, Japan; Dassault Systemes BIOVIA, UNITED STATES

Scientific conferences are one of the most common venues for researchers and others to present and exchange new findings. In recent years, “unconferences”—i.e., meetings that promote spontaneous discussions rather than predetermined presentations—have emerged as a more collaborative approach. Unconferences differ from conferences in key ways: while conferences have a predefined set of presenters together with an audience, unconferences promote more collaborative and spontaneous interactions across participants [1]. A “hackathon” is a special kind of unconference in which people come together to state, discuss, and solve problems by means of collaborative brainstorming, modeling, design, coding, testing, and documenting [2]. Despite the “hack” portion in the term, hackathons welcome not only software developers but anyone involved in creating solutions that can be later consumed or exposed via software. In addition to collaboration, hackathons promote community development around a subject used as the main hackathon topic. Such a topic can correspond to a knowledge domain (e.g., semantics or genomics) or be related to a particular organization (e.g., data and data services offered). There are hackathon-like events that are called by other names, such as “codefests.” In this paper, we include such events under the umbrella term “hackathon.”

Hackathons are especially useful in bringing together interdisciplinary sets of domain experts and specialized computer scientists with various degrees of experience and skills to “hack” solutions related to scientific topics of mutual interest. While traditional conferences focus more on transferring knowledge, hackathons are more about collaboratively generating solutions. The interactions often lead to productive collaborations, professional development opportunities, and a network of resources. Well-run hackathons are very effective at building a community among participants. Hackathons provide an opportunity for researchers and developers to interact and brainstorm with other participants in an appropriate environment to accelerate collaborations on topics of mutual benefit. In addition, they can provide a unique opportunity to think through a problem, without the usual distractions. Therefore, hackathons can be very productive and result in a major impact on the targeted topic and/or community [3]. However, organizing a successful large-scale hackathon takes significant time and effort; e.g., organizing committees for the National Bioscience Database Center (NBDC) [4]/Database Center for Life Science (DBCLS) [5] and the ELIXIR Europe BioHackathons start approximately 1 year in advance.

We use the term BioHackathon to refer to those hackathons addressing problems in domains related to biomedical and life sciences. BioHackathons are recognized as having a growing impact and purpose within the life sciences community. Some of these events have been running for more than a decade; e.g., the first BioHackathon was organized by the Open Bioinformatics Foundation (OBF) [6] in 2002, followed by the NBDC/DBCLS BioHackathon series, the National Evolutionary Synthesis Center (NESCent) hackathon series [2], and the OpenBio Codefest event organized by the OBF and usually held right before the annual Bioinformatics Open Source Conference (BOSC) meeting. In recent years, we have seen an increasing number of these events running independently or as a part of traditional conferences. For instance, within only the area of Cambridgeshire in the United Kingdom, 2 of the main life sciences institutes in the area have organized BioHackathons or similar coding events during the last 2 years (2018 and 2019): the Sanger Wellcome Trust Institute [7] and the European Molecular Biology Laboratory (EMBL)-European Bioinformatics Institute (EBI) [8]. In the United States, National Center for Biotechnology Information (NCBI) “codeathons” have been actively hosted [9]. Many of these hackathons and their products are being compiled in a list [10].

What follows is mainly a compilation of our experiences organizing and attending the NBDC/DBCLS BioHackathon series [11] as well as the first ELIXIR BioHackathon [12]. The idea of compiling a list of guidelines for running BioHackathons started as a NBDC/DBCLS BioHackathon project in 2017. As commonly happens with BioHackathon projects, someone suggested the idea, and those interested signed up to work on it—a collaborative process from start to end. To structure our suggestions as a “Ten simple rules” manuscript, we followed the recommended approach for writing this kind of paper [13]. We also took into account other similar events, such as the Semantic Web Application and Tools for Health Care and Life Sciences (SWAT4HCLS) [14] hackathon, the Stanford Health Hackathon [15], codefests organized by the OBF [16], and the Sanger Wellcome Trust hackathon [7]. This list of 10 guidelines for running an impactful large-scale hackathon is a distillation of the authors’ collective experience attending and helping organize many such events. We present here 10 simple rules related to prehackathon, hackathon, and posthackathon activities. A summary is presented in Fig 1.

**Fig 1:**
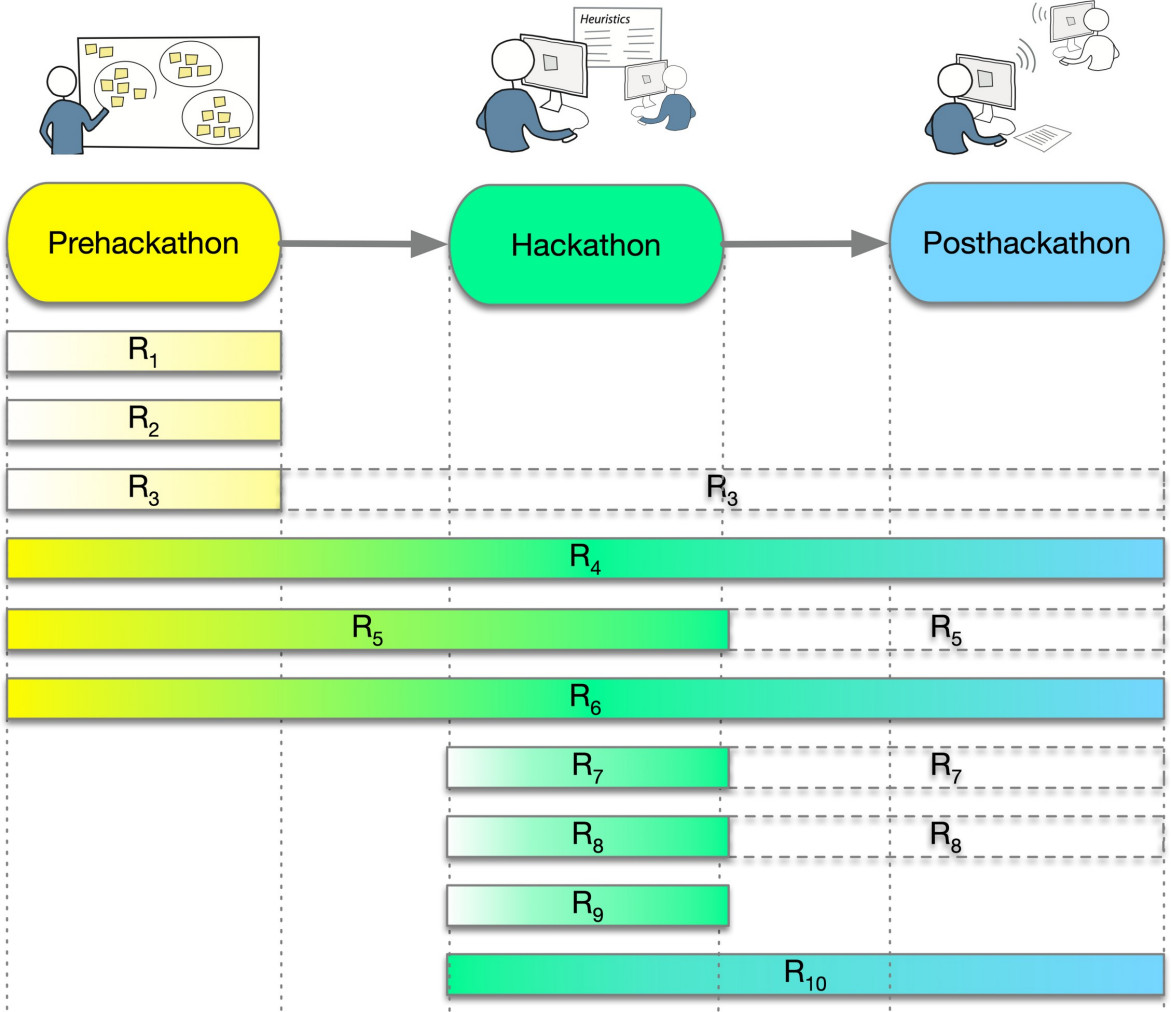
A graphical overview of 10 simple rules to successfully run a BioHackathon. The rules, denoted by R_i_ (in which *i* is 1 …10), are expected to be applied in particular phases: prehackathon, hackathon, and posthackathon. Certain rules are valid over more than one phase or the entire process (e.g., R_6_), whereas R_3_, R_5_, R_7_, and R_8_ can encompass multiple phases (denoted in the figure by dotted boxes).

## Rule 1: Define the goals, scope, and team

A well-defined goal should set the scene. It will give you focus and help you decide on the scope of your BioHackathon. As an organizer, you should try to define expected outcomes: whether improving the coverage of your data services, advancing knowledge on a particular domain via software development, or answering very specific questions. Defining expected outcomes can also help you home in on topics to work on during the hackathon. You also want to take the perspective of the attendees and think about their expectations. People commonly attend unconferences to interact with others and learn useful information or skills [1]. In the case of BioHackathons, participants also want to transform ideas into models, designs, and programs that can later improve and extend the status quo.

A clear goal will not only guide you as an organizer but will also make it easier for potential attendees to get a quick idea of what kind of projects they can participate in. The goal is a “lens” you can use to focus efforts and define a scope, including elements such as audience and duration. If it is the first time you are running a BioHackathon on the selected topic, you will need to do some research to gauge the interest that it will raise within the targeted community. This will later help you define the kind of venue you need as well as ensure a nice balance between senior and junior participants.

Organizing a BioHackathon is not a one-person activity. The initial idea can come directly from you or from discussions with colleagues; however, once you have decided to go for it, you need a team with defined roles and rules [17]. You will need people to communicate with funders, sponsors, venues, and travel agencies. Make sure you have enough organizers to “cook” (but not so many that they burn down the kitchen) and one master chef coordinating it all. From our experience, you will need the following: one chair and, optionally, one vice-chair; one or two people in charge of finding the venue and organizing things related to it; one or two people in charge of the website and communication channels (e.g., common files on a repository and/or Google Drive, Twitter [18], or messaging systems); and one or two people looking for local sponsors (if needed). In addition, if you have decided to have a call for projects, you will also need a group of people to review and assess those. The number of people in that committee largely depends on the number of applications you are expecting; we suggest between 10 and 15 people on the committee for a hackathon expecting approximately 100 participants.

## Rule 2: Find the right venue

You have defined a goal and a scope. You have an idea of the number of participants. You know your budget. It is time to find the right venue for your event. The budget will have a high impact on the venue options; however, it does not necessarily define the kind of hackathon you will run. The earlier you can reserve the space the better. This will give you the opportunity to negotiate a good deal and prepare yourself for the event. It will also give you the chance to find a date not overlapping with other similar events or major conferences within the same domain [1].

For a BioHackathon, you want to find a venue that supports interaction, discussions, and software development, from brainstorming to testing. The venue should be big enough to host all participants and offer a comfortable and relaxing atmosphere and spaces to clear your mind. Go for an open space where people can organize in hackathon groups and freely move around but also consider providing smaller rooms for discussions without disturbing others. Catering should also be carried out at the venue so that meals become an opportunity to interact with each other. Whether you go for formal or informal catering depends on the size of your hackathon. The larger the event is, the more help you will need. If possible and affordable, we recommend organizing catering directly with the venue.

Depending on the topics and the number of attendees, you will need to consider (at the minimum) the following:

- Dedicated high-speed internet that is stable and has enough bandwidth; a backup internet line is advisable (as it can be easy to overwhelm the internet at a venue)
- Adequate electrical grid support, as more computers (and devices) than usual will be connected
- A large room so that everybody can comfortably fit and still have enough space for grouping, discussing, and coding; ideally, the space can remain open until late night
- Smaller rooms for project team meetings
- A location away from distractions (including tourist attractions) but with spaces to clear your mind (e.g., seaside)
- Enough extra spaces to promote networking and spontaneity
- Appropriate supplies, including tables, seats, power strips, projectors, microphones, sticky notes, color markers, notepads, pens, and pencils
- A reception area where you distribute name badges and provide logistics information (e.g., transportation)
- Drinks (e.g., coffee, tea, water) and snacks available at all times

## Rule 3: Ensure balanced and representative participation

In regard to hackathons, you have 3 main options: (i) everybody is welcome as long as there is space, (ii) participants are somehow selected in order to ensure a representative and balanced participation, and (iii) a mix of the previous 2 options. Although it requires more planning, we recommend option 2 or 3. A call for participation in which you can learn more about the projects and interests of participants will help you not only find this balance but also achieve your goals. This may be even more important if you are fully or partially covering expenses for some of the participants. If there is still available space, then you can open the event to other interested participants. Make sure you provide a clear and transparent selection procedure as part of your call.

There are multiple platforms that can facilitate a call for participation and registration. Use them, because they will make your life easier and will allow people to provide useful information regarding interests, skills, dietary preferences, and allergies. The call for proposals, selection, and registration, combined with your website, will establish your initial contact with participants. We understand that you would want to cover as many participants as possible; however, budgets and space at venues are finite. Hosting BioHackathon projects within open repositories such as GitHub will reach audiences beyond the hackathon participants (see Rules 6 and 10). In this way, BioHackathons also contribute to open and collaborative science.

Aspects considered for balanced participation can include, for instance, a range of expertise levels (from junior to senior), local and international participants, and gender balance. A discussion of how to achieve gender balance goes beyond the scope of this paper; it would probably require its own set of 10 simple rules. In short, being conscious about gender balance and looking for ways to achieve it is a good start [19]. Leading by example is a common strategy [20], so make sure that these considerations for balance are an important and visible part of your agenda [21]. Appoint women (and people from other underrepresented groups) to leadership positions at your event; try to ensure that project groups are not homogeneous so that everyone benefits from having a variety of perspectives.

BioHackathons running for multiple days are commonly project oriented, meaning that there is an initial selection of projects together with the first batch of participants, i.e., project submitters. Once the main projects have been selected, additional participants can freely register. One-day BioHackathons—either solo or collocated with another event such a conference—are quite open in terms of participation and usually welcome anyone interested on a “first come first served” basis. If you are running a project-selection process, in general terms, you want to include diverse but also compatible projects so that people can collaborate together and learn from each other, boosting not only what is accomplished but also the participants’ experience, moving from a couple of days of interaction to longer professional collaborations and even friendships. Make sure you select a good variety of projects across the different activities related to problem-solving—from brainstorming to testing—thus facilitating different technical levels of participation. Additionally, include challenging projects, requiring different levels of expertise, and possibly an interdisciplinary approach. These sorts of projects will attract junior and senior participation as well.

Depending on the location, getting to the venue may be expensive for international participants. In any case, you will get a high number of local participants because promoting science in your region is probably one of your goals, and it is easier and cheaper for locals to attend. Nevertheless, you want a reasonable number of international participants to promote a good exchange of new and different ideas, improving the chances of collaboration beyond your BioHackathon.

## Rule 4: Compile and regularly review your checklist

You will need at least 3 main checklists—that include activities and times—for prehackathon, hackathon, and posthackathon organization. These lists should interconnect to each other. While we cannot tell you what to include on your final checklist, we can mention some common things to keep in mind. You will notice that some of the items correspond to rules in this document:

- Prehackathon
  ○ Website (ideally with a permanent URL, e.g., www.biohackathon.org)
  ○ Call for participation
  ○ Registration
  ○ Hotel and flight bookings
  ○ Special requests—e.g., dietary requirements—from participants as well as a cancellation policy
  ○ Logistics regarding, for instance, transportation, catering, and memorabilia
  ○ Communication channels (e.g., typically email)
  ○ Wi-Fi, power cables, and general supply acquisition and transport
- Hackathon
  ○ Hackathon room
  ○ Logistics regarding presentations, discussion, and code repository
  ○ Regular announcements and updates
  ○ Communication channels
- Posthackathon
  ○ Wrap-up, reports (e.g., a “writathon”), and potential publications
  ○ Communication channels
  ○ Keep paying for your website domain to avoid hijacking
  ○ Encourage participants to continue collaborating

## Rule 5: Create and maintain effective communication channels

There are plenty of communication platforms; some are more useful than others for hackathons. Here, we will mention some of the most popular ones. To start with, as mentioned in Rule 4, you will need a registration system as well as a website with a persistent and easy-to-recall domain. While creating a domain name, you should keep in mind that the hackathon might become a series; thus, it is not advisable to capture the year in the domain name, as it will not be sustainable (e.g., http://www.biohackathon.org instead of http://www.biohackathon2019.org). Instead, you can use a subdomain (e.g., http://2019.biohackathon.org/) or a local path (e.g., http://www.biohackathon.org/2019/) to host a website for the specific event of a series.

To advertise your event, you can use mailing lists, social media, and academic and professional networks. In regard to social media, make sure others can easily identify you. Create, as an organizer, a Twitter handle and hashtag for your hackathon so that people can remember it and associate it with your event. A dedicated, moderated mailing list for your BioHackathon will help you to easily communicate with all attendees at once and will also allow them to interact with each other from early to late stages. Remember to encourage all participants to join and find ways to avoid spam as much as possible. Create a shared space where people can host ideas, discussions, and code—our recommendation for that is GitHub—with the advantage that open repositories will also make all of the BioHackathon results available to the whole scientific community. Use an instant messaging system so people can easily interact across teams during the hackathon phase. For these, Slack [22] and Gitter [23] are popular ones for working collaboratively. Not all participants will use all of the created channels, so replicate information across them for those highly important points you want to broadcast.

Once you have created the different channels, make sure you keep them updated. You cannot be certain which ones will be preferred by participants, so try to cover multiple fronts without flooding participants with information. In summary, we find the following communication channels useful.

### A website

Use this channel for official information that needs to be easily accessible at any time, such as the following: how to reach the organizers and how to participate together with a list of communication channels; an overview of the process, from the call for participation to posthackathon activities; important dates and deadlines, as well as information on the venue and how to get there; if allowed by attendees, a list that includes some basic information such as affiliation; and a code of conduct and procedure to raise issues. One important aspect that you should make clear from the beginning is the collaborative nature of BioHackathons; make sure that everybody understands that the projects are open for participation. Additionally, if you plan to make the resulting products of each project open to the public, a policy for credits and licenses should be described.

### A general mailing list (as well as an internal mailing list for the organizing committee)

Use this channel for announcements. Make sure the mailing list is searchable so that newcomers can still access previous conversation threads. Always remember to allow enough time if the announcement requires some reaction from recipients. Together with your website, these will be your main communication channels.

### Twitter

Use this channel to advertise and promote your event and reach a broader community. Make public the handle and hashtag that you want people to use when referring to the event. You can also reinforce important information, such as milestones or deadlines, by Tweeting about it. Although Twitter is a common social media platform, not everybody uses it, so this should be seen as a secondary rather than a primary channel.

### Instant messaging

Use this channel for quick communication closer to the BioHackathon dates and during the event. Promote this channel as a means to facilitate communication within and across project teams. During the event, make sure that important information is also communicated face to face such as via a microphone.

### Code repositories

Use this kind of channel to host activities related to projects even before the BioHackathon starts. Encourage participants to include here not only the code but also the goals, participants, discussions, diagrams, models, etc.

## Rule 6: Promote a welcoming and respectful environment

So far, we have covered some logistics, participant selection, and communication channels. All of this should be framed by a welcoming and respectful environment in which people feel safe and included regardless of their differences. Providing a code of conduct, together with the channels to communicate any irregularities, is key for any event. If you do not have a code of conduct, you can adapt those that other, similar events have implemented; either a template [24] or a fully developed code of conduct [25] can be a good starting point. For example, international participants may encounter cultural factors that give rise to unintentional issues. Encourage participants to speak up whenever they perceive disrespectful, bullying, harassing, or discriminatory attitudes.

In a similar vein, people also need to feel safe and respected regarding their intellectual contributions. It should be clear from the beginning who will own the intellectual property (IP) rights to the ideas and solutions discussed and coded during the BioHackathon. As an ideal space to boost collaborations, BioHackathons should, as much as possible, opt for open-source code projects hosted in public repositories, where participants retain the ownership and IP rights of their own work. Appropriate mechanisms should be used by participants to ensure that any previous work, including data, software, or any other digital object, is rightly credited to the original authors and used within their license scope. It is important for any piece of software to define clear and transparent contribution, governance, and communication processes [26]. Such a recommendation can be extended to any digital object, given the different aspects of problem-solving covered at BioHackathons. Regardless of who retains the IP rights, the role of the BioHackathon should be acknowledged in any future developments or publications.

## Rule 7: Encourage team-building, self-organization, and cross-collaboration

If you have opted for a call for projects, you will know beforehand most of the projects that will be developed during the BioHackathon. Even if you have not, from the event registration you will discover interests and skills from participants. Use this information, together with your goals, to suggest hackathon teams covering one or more related projects. These teams should promote cross-collaboration while helping avoid silos. Organize the hackathon rooms and/or tables to make it easier for everyone to know where teams are sitting together. Let attendants decide themselves which team they want to join or whether they want to create new teams. Invite teams to (i) designate a team leader promoting organization and collaboration within the group and (ii) advertise their activities via common code repositories allowing others to learn about what each team is working on and thus promote cross-collaboration. Make sure all participants understand that they can contribute to multiple teams and freely move from one team to another. For instance, a participant can choose one particular team as her/his main base but still participate in brainstorming, discussion, or testing activities carried out by other teams. Movement is natural in BioHackathons lasting a couple of days as people will find other groups to collaborate with or find exciting new topics to learn about.

In addition to announcing the hackathon projects on your website, you will need a kick-off session, during which proponents pitch the projects to the other participants. This makes it easier for everybody to identify the available people and subjects. A prehackathon activity is useful to this end. This prehackathon activity can take the form of a symposium on the first day of the event (if your BioHackathon is running for multiple days) or a kick-off meeting (if you do not have much time). Keep a balance, because you want to show a variety of projects so that the full spectrum of subjects is covered but do not want to overwhelm the audience with too many diverse talks. It is recommended that you provide a space for newcomers to introduce themselves as well as their interests, e.g., via short presentations. Make sure that presenters know how long they have, what the presentation order is, and where their slides should be uploaded prior to the event. You will also need to introduce basic logistics, e.g., where the toilets and emergency exits are, the teams you have already identified, where the teams are gathering, and what the agenda is. This is particularly important if you plan to have retrospective sessions and written hackathon reports because people will need to prepare for those. It is recommended that you record (video) the symposium presentations so that people who could not attend the talks can catch up later. The NBDC/DBCLS BioHackathon has been sharing those videos via a YouTube channel.

As an example of how a hackathon can be organized, the NBDC/DBCLS BioHackathon includes a 1-day symposium, 5 days for hackathon activities, and half a day for writing team reports and wrapping up the hackathon. Hackathon teams are announced or self-organized during the first hackathon day and are categorized around typically cutting-edge subjects (e.g., genomics or glycomics) and semantic technologies (e.g., data integration or machine learning). At the end of the third hackathon day, the teams present their progress reports in the form of flash presentations. The ELIXIR BioHackathon includes a half-day symposium, 3 days for hackathon activities, and half a day for team report writing and wrapping up. A midterm team report session was held on the second hackathon day during 2018, whereas daily reports were used for the 2019 version. Hackathon teams are organized slightly differently, as they take into account ELIXIR platforms, flagship projects, and communities. Your team building can vary according to your goals and expectations. If possible, try to have a similar size across teams and always allow the creation of last-minute teams. Some teams will move from one BioHackathon edition to the next; if that happens, make sure newcomers feel welcome as part of those existing teams. Rule 3 (“Ensure balanced and representative participation”), together with Rule 6 (“Promote a welcoming and respectful environment”), should help here.

Even before the BioHackathon starts, and if the European Union (EU) General Data Protection Regulation (GDPR) [27] allows it, you might want to share a list of participants, projects, and skills so they can identify common interests. If many people are travelling from a distance to the event, communication channels can also boost encounters at airports and transport sharing. All of this will promote team-building and cross-collaboration from day 0.

## Rule 8: Provide informal exchange spaces

Well-organized and productive hackathons maximize the possibility of meeting other participants with different expertise. Having a fixed location for each project team throughout the hackathon can make it easier for attendees to find and talk with people from a particular team of interest. The ideal atmosphere should be relaxed, open, friendly, respectful, and fun [1]. Even if people bring their own project, there are different approaches to follow and technologies to work with. Providing informal exchange spaces—from a simple space for coffee, tea, and biscuits to space for more specialized ice-breaking activities—can help boost collaborations that will extend beyond the days of the hackathon. It may be that people will not end up joining other teams, and yet they will become aware of what others are working on and will find an opportunity to cross-pollinate by contributing ideas, thoughts, and considerations to multiple groups.

A relaxed and respectful environment will also make it easier for junior participants to comfortably approach senior participants and for newcomers to talk to regular attendees. You can also help break the ice with some extracurricular activities. Some venues, particularly those specialized in team retreats, will offer assistance with such activities; keep in mind that this may come at an extra cost. Another possibility is to do your own research, regarding not only ice-breaking activities but also networking. Networking can be facilitated by encouraging attendees to express their interests to others, asking questions, and talking to people they have never talked to before. One way to mix ice-breaking and networking sessions is a wall with sticky notes where people can express their expectations, how the day went, what they learned, or what help they needed; you can use columns for what you want to capture and colors to express emotions. Use your imagination but be pragmatic, use the extra activities to boost networking but also productivity. In this way, you are promoting an exchange of ideas, expertise, and knowledge. Remember, from Rule 1, that people attend unconferences to interact with others but also to learn useful information or skills. This is one of the reasons why we recommend representative and balanced participation (see Rule 3). The more interaction there is, the greater the exchange of ideas, the more team-building, the more coding, the more success, and the more chances your event will have a meaningful impact.

Keep in mind that hackers also need to rest in order to be productive. While machines are used to hack, it is people who are operating those machines. People get tired after intensive workdays, and people get grumpy when things are not going as planned (or as quickly or simply as they thought they would). Introducing spaces for some entertainment and socialization, especially if your event runs for a couple of days, is a good idea. Karaoke, table football, ping-pong, and video games are some possibilities to explore. Depending on your budget and if time allows, you can also reserve a couple of hours for a social activity outside the hackathon venue, e.g., rowing on a lake close by or visiting a museum. Keep in mind that not everybody will want to participate in these activities; offer the possibility, but let people decide what works best for them without feeling left aside. For a 1-day hackathon, such a socialization space will probably be just the coffee break, lunch, or dinner.

## Rule 9: Include retrospective sessions along with documentation

A moment to recapitulate your steps and share progress with others will provide a refreshing pause from the hackathon and will keep everybody aware of what others are doing. Depending on the duration of your BioHackathon, you can have both a midterm pause and a wrap-up session or have only the latter. This also encourages people to keep discussions alive and to document it all while simultaneously avoiding isolation. A retrospective session is not as informal as a coffee or tea break but still promotes the sharing and exchanging of ideas with others. If your posthackathon plan includes writing a report of the event, this will also become an opportunity for people to add words to their projects beyond the coding aspect.

While the hackathon tasks are very active, the retrospective sessions tend to be more passive, as there will be a main speaker and people listening. To make it easier for everybody, encourage short reports, be strict about the time, and suggest a template so that everybody focuses on the key aspects to be presented, e.g., achievements so far, what is still missing, and any help needed. Give an option to use a demo or video instead of slides. Regardless of the presentation approach, use a common repository for all presentations so you can keep information centralized. This will help later in case the organizers or participants want to use these presentations for further communication beyond the event or more formal publications about the projects. You can also ask for comments regarding how happy people are with the event or what exciting things happened to them during the hackathon. This may elicit some funny comments that will recapture interests from the audience. If the retrospective takes more than a couple of hours, include a formal coffee break so people can also stand and move a bit. A report wall is a good alternative for short hackathons (e.g., 1-day or 2-day hackathons) [28]. Rather than having a formal report session, ask participants to document their discussions and achievements via printouts or sticky notes on a wall. Remember to collect that material and take a photo of the display so that you can easily access the recorded information later.

It is always important to document the activities carried out during the hackathon sessions within the different hackathon teams. In Rule 7, while talking about teams, we mentioned the creation of a team code repository. Such a repository should be used as a project logbook so that all activities—including expectations, challenges, failures, successes, and outcomes—are documented and freely available to everybody, even outside the hackathon. Even if you are not working on an open-source code project (some companies or sensitive data may prevent doing so), in order to make it findable and accessible, metadata should be provided (e.g., domain type such as proteomics). Documentation, which is often neglected, is an important piece complementing the metadata. Gently remind and encourage attendees to document it all as much as possible. For instance, organizing committee members can use social communication channels to let people know about what challenges they have faced and where the solution has been documented; others can then copy the idea. If this is not the first time you are running your BioHackathon, a mention of the publications related to projects from previous versions (and how some of them came from documents developed during the BioHackathon) can encourage attendees to better document their projects.

## Rule 10: Plan the next steps

Even if it is a one-time hackathon, you will want to know what has gone well and what has not. Getting feedback is the minimum amount of follow-up you want to conduct. If the hackathon is part of a series, your next steps will start all over again as planning for an improved version of your BioHackathon begins. Gather with the organizing team to recapitulate what went well, what did not, what you want to repeat, and what you want to avoid. Keep your communication channels open so people can reach you beyond the event; they can contribute with useful ideas on how to improve for the next time.

In addition to gathering feedback and recapitulating lessons learned, we recommend that you report on the activities carried out during the hackathon. Options such as preprints and peer-review journals are available; any option is good, as long as you share your story with the scientific community. To cite an example, the NBDC/DBCLS BioHackathon has regularly published its outcomes in various journals. The first paper was published in 2010 [29], while the last was submitted in early 2019. Reporting on hackathon accomplishments is not straightforward. Some preprint servers and many peer-reviewed journals do not accept this type of publication, since hackathon projects are often ongoing work. In response to this challenge, the NBDC/DBCLS BioHackathon community has created a BioHackathon preprint server, BioHackrXiv, launched at the beginning of 2020 as part of the Open Science Framework (OSF) preprint collection [30]. Other possibilities include journals specializing in data, software, or research objects. If you are using GitHub, remember that code can be released via Zenodo [31]. In this way, you can reach a broader audience, far beyond your BioHackathon. This will result in the lasting impact of your event [17] and will contribute information back to the scientific community, ultimately advancing knowledge. Remember to let your BioHackathon attendees know how to acknowledge the event in any publication that comes from the work started, advanced, or finished during the BioHackathon.

## Final thoughts

BioHackathons are usually driven by community problems; very often these are part of international research endeavors deeply rooted within existing communities of practice. Hackathon provides an effective and productive environment to promote collaboration for solving problems then and there. Additionally, the flexible nature of hackathons often results in the emergence of new ideas and teams that target next scientific challenges. The solutions coming from hackathons are continued and moved forward by these communities, helping advance life sciences and open-source research [3]. Such outcomes are sometimes hard to report in a timely manner and are thus not sufficiently discoverable, but this situation will be improved by the newly launched preprint service, BioHackrXiv [30], which is also a product of a BioHackathon. Here, we have compiled a list of what, based on our experience as organizers and attendees, some of the main rules to successfully run a BioHackathon are. Some of these rules are shared with those related to scientific meetings [17], some others with unconferences [1]. We have focused on and extended those that we see as more relevant for BioHackathons.
